# A smart mask for active defense against airborne pathogens

**DOI:** 10.1038/s41598-021-99150-x

**Published:** 2021-10-07

**Authors:** Rohan Reddy Kalavakonda, Naren Vikram Raj Masna, Soumyajit Mandal, Swarup Bhunia

**Affiliations:** Electrical and Computer Engineering Department, University of Florida, Gainesville, FL 32611 USA

**Keywords:** Electrical and electronic engineering, Quality of life, Lifestyle modification

## Abstract

Face masks are a primary preventive measure against airborne pathogens. Thus, they have become one of the keys to controlling the spread of the COVID-19 virus. Common examples, including N95 masks, surgical masks, and face coverings, are passive devices that minimize the spread of suspended pathogens by inserting an aerosol-filtering barrier between the user’s nasal and oral cavities and the environment. However, the filtering process does not adapt to changing pathogen levels or other environmental factors, which reduces its effectiveness in real-world scenarios. This paper addresses the limitations of passive masks by proposing ADAPT, a smart IoT-enabled “active mask”. This wearable device contains a real-time closed-loop control system that senses airborne particles of different sizes near the mask by using an on-board particulate matter (PM) sensor. It then intelligently mitigates the threat by using mist spray, generated by a piezoelectric actuator, to load nearby aerosol particles such that they rapidly fall to the ground. The system is controlled by an on-board micro-controller unit that collects sensor data, analyzes it, and activates the mist generator as necessary. A custom smartphone application enables the user to remotely control the device and also receive real-time alerts related to recharging, refilling, and/or decontamination of the mask before reuse. Experimental results on a working prototype confirm that aerosol clouds rapidly fall to the ground when the mask is activated, thus significantly reducing PM counts near the user. Also, usage of the mask significantly increases local relative humidity levels.

## Introduction

The onset of the SARS-CoV-2 pandemic in late 2019 saw an immediate adoption of preventive measures to control the airborne spread of this virus^1^. Citizens worldwide were advised to take precautions such as social distancing, face coverings, and good hygiene practices, while governments adopted technologically advanced measures like contact tracing. Face coverings are effective in reducing the spread of the virus, even with high levels of exposure in closed spaces^2^. However, existing face masks do not adapt to changing exposure levels, i.e., only provide passive protection^3^. As a result, they are inefficient when these levels are unpredictable and fluctuate over time, which is generally the case. Thus, there is a need for smart protection mechanisms that not only act as physical barriers but also use active mitigation techniques to reduce the spread of pathogens whenever necessary.

**Figure 1:**
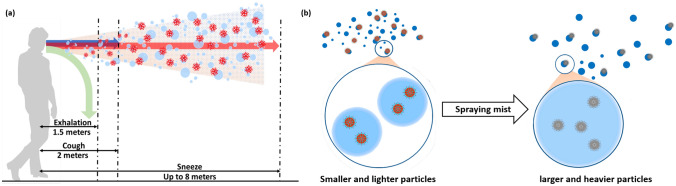
(**a**) Droplets expelled from mouth during various activities like exhalation, coughing, and sneezing create aerosol clouds that travel to different distances. (**b**) Expelled droplets containing suspended virus particles are small and easily turn into aerosols (left). The proposed ADAPT smart mask sprays mist on these particles (right), which loads them (i.e., increases their size and mass) and thus causes them to rapidly fall to the ground.

Regular human activities such as breathing, talking, sneezing, and coughing discharge droplets into the surrounding environment, creating a cloud of aerosol particles (Fig. 1a)^4^. For example, a single sneeze^5,6^ produces as many as 40,000 droplets with diameters between 0.5–$12\,\mu \hbox {m}$ that are expelled at speeds up to 100 m/s and can reach distances up to 8 m. Similarly, a single cough produces up to 3000 droplet nuclei, and comparable numbers are typically recorded after 5 min of talking. Such aerosol clouds include various types of cells (e.g., epithelial cells and leukocytes), ions present in mucus and saliva (e.g., Na$+$, K$+$, and Cl−), and, potentially, various infectious agents suspended in the droplets (e.g., bacteria, fungi, and viruses). Even weak air currents caused by innocuous daily activities, such as walking^7^ or opening doors, can transport these infectious droplets over long distances ($>10\,\hbox {m}$). Such airborne transmission is the primary driver for virus spread in indoor spaces such as bathrooms, doctor’s offices, daycare centers, and public transportation; it is also important in many outdoor environments^8^.

The SARS-CoV-2 virus is 100–120 nm in diameter and can remain suspended within droplets of diameter $d>0.2\,\mu \hbox {m}$^9^. Droplets with $d>5\,\mu \hbox {m}$ fall to the ground quickly due to gravity, while very small droplets evaporate^10^ and aerosolize in a few seconds to droplet nuclei with $d\approx 1\,\mu \hbox {m}$^11^. Fortunately, most masks can filter out droplets of this size: many materials have $\ge 96\%$ filtration efficacy for particles $>0.3\,\mu \hbox {m}$, including 600 TPI (threads per inch) cotton, cotton quilts, and cotton layered with chiffon, silk, or flannel^12^. Thus, the most critical requirement for the proposed closed-loop smart mask is to eliminate the small (but potentially significant) fraction of virus-laden droplets that are $<0.3\,\mu \hbox {m}$ in diameter.

Unlike existing masks, the proposed system actively detects the presence of nearby airborne particulate matter (PM) that can contain viruses or other pathogens. It then analyzes the sensor data (including concentration, size distribution, and other PM properties) to determine a smart mitigation strategy that minimizes aerosol spread. In our experimental prototype, mitigation is provided by a cold mist generator that loads the particles, which increases their aerodynamic diameter and mass and makes them quickly fall to the ground (Fig. 1b). The settling time $t_{s}$ of aerosols scales as $d^{-2}$, making the proposed mitigation method particularly effective for small particles ($d<0.3$ $\mu$m) that are not efficiently filtered by masks. For example, the value of $t_{s}$ in both still and turbulent air decreases from $\approx 130$ hours for $d=0.3$ $\mu$m to only $\approx 8.2$ min for $d=10$ $\mu$m^13,14^. However, all particles of a given size have the same settling time in still air, while in turbulent air the probability of settling increases exponentially with time, i.e., as $p(t)=1-\exp (-t/t_s)$. Additionally, the mist spray creates an airflow pattern that actively blows droplets away from the user. Adaptability is provided by algorithms running on the on-board controller that adjust the mist generator’s spray angle, intensity, and duration based on sensor data. Such active closed-loop monitoring and protection can remove viruses (and other pathogens) from the air before they can infect others, thus reducing the need for periodic disinfection of the area while also providing increased protection to both the wearer and others in the vicinity. In fact, groups of smart mask users can collaborate to increase the local relative humidity (RH), which in turn reduces the probability of airborne transmission^15^. Specifically, increased RH allows nasal mucus to more easily carry airborne pathogens to the stomach where they are destroyed by digestive acids.

**Figure 2:**
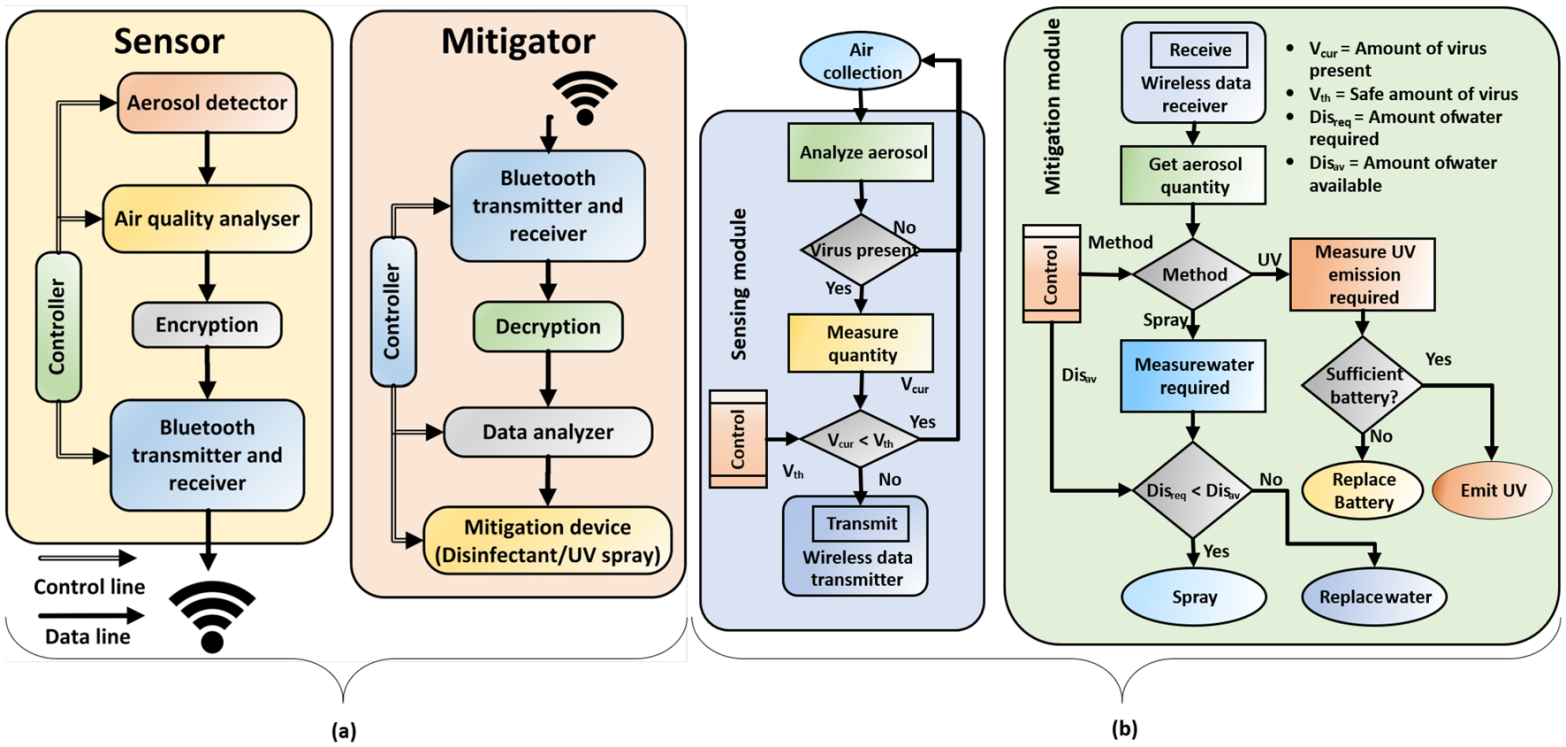
(**a**) Block diagram of the data and control structure of the sensing and mitigation modules in the ADAPT smart mask. (**b**) Flowchart of the sensing and mitigation algorithm used during closed-loop operation of the mask.

The proposed smart mask contains two main hardware modules: the PM sensor and the active mitigation device. Standard PM sensing techniques include impactors and filters^16^ for collection, and real-time polymerase chain reaction (RT-PCR)^17^ or surface-enhanced Raman spectroscopy (SERS)^18^ for analysis and identification. However, it is difficult to implement such techniques in real-time within a portable form factor and limited power budget. Thus, we employ a simpler detection approach based on laser scattering. Specifically, the PM sensor (SPS30, Sensirion) detects scattered laser light to count airborne particles and categorizes them in “bins” based on mass concentration ($\mu$g/$\hbox {m}^3$) and number concentration (1/$\hbox {cm}^3$). Since aerosol travel distance also depends on environmental factors such as RH and temperature^16^, the on-board control algorithm combines data from PM and environmental sensors to determine air quality in real-time. The proposed algorithm (Fig. 2a) classifies air quality based on health risk (e.g., “very high,”, “high”, “moderate”, and “low”). The output categories are then encrypted for security and sent to the mitigation module via either a wired or wireless (e.g., Bluetooth) connection. A “high risk” output triggers the mitigation module (Fig. 2b), which generates aerosolized mist on-demand using a piezoelectric transducer. The smart mask can also connect to authorized mobile devices through a Bluetooth module. A custom mobile application allows users to monitor current air quality, check system status (e.g., battery life, liquid level, notifications, and alerts), and manually override the on-board automated mitigation algorithm if desired.

While our prototype uses tap water to generate the mist spray, in general a variety of liquids, colloids, or solid suspensions can be used. For example, solid additives can increase droplet density and/or size (thereby decreasing $t_s$), while disinfectants can actively kill pathogens. The best disinfectant for a given pathogen can be found using guidelines provided by agencies such as the U.S. Centers for Disease Control and Prevention (CDC)^19^; common choices include diluted bleach, soap, and $>70\%$ alcohol solution. However, since the safety of such disinfectants when inhaled is unknown, their use will be studied in future work. Furthermore, later versions of the device can be equipped with machine learning (ML) algorithms that learn when respiratory droplets are likely to be present in a location and proactively employ the proposed active mitigation mechanism.

## Results

We tested a prototype of the ADAPT smart mask in various scenarios. Our experiments confirm the expected increase in particle sizes and decrease in settling time when the mask is turned ON. We also tested our mitigation module with several water-based solutions. Finally, we verified the ability of the mask to increase local RH.

### Effects of ADAPT on aerosol concentrations

We emulated the scenario of a person wearing an ADAPT smart mask by mounting it at a height of $\approx 1.6\,\hbox {m}$ inside an enclosed environment with no significant air currents. The smart mitigation module used water to generate the mist spray, while a separate humidifier generated water droplets to emulate a potentially-infectious aerosol cloud. Finally, PM sensors were used to measure particle counts and sizes in the region around the mask.

Two PM sensors were used to compare aerosol properties with and without the smart mask. One of the sensors (“int”) was mounted on the mask along with the mitigation module, while the other (“ext”) was placed on the ground. Initially, the humidifier was turned ON for 15 sec with the mitigation module turned OFF. The resulting outputs of both PM sensors (local number and mass concentrations) were monitored until most aerosols had settled out ($\approx 160\,\hbox {s}$). Next, the tests were repeated with the mitigation module turned ON but the humidifier turned OFF. Finally, the effectiveness of the smart mask was verified by turning ON both the humidifier (for 15 sec) and the mitigation module (for 15 sec after the PM sensor in the mask detects a significant change in concentration).

**Figure 3:**
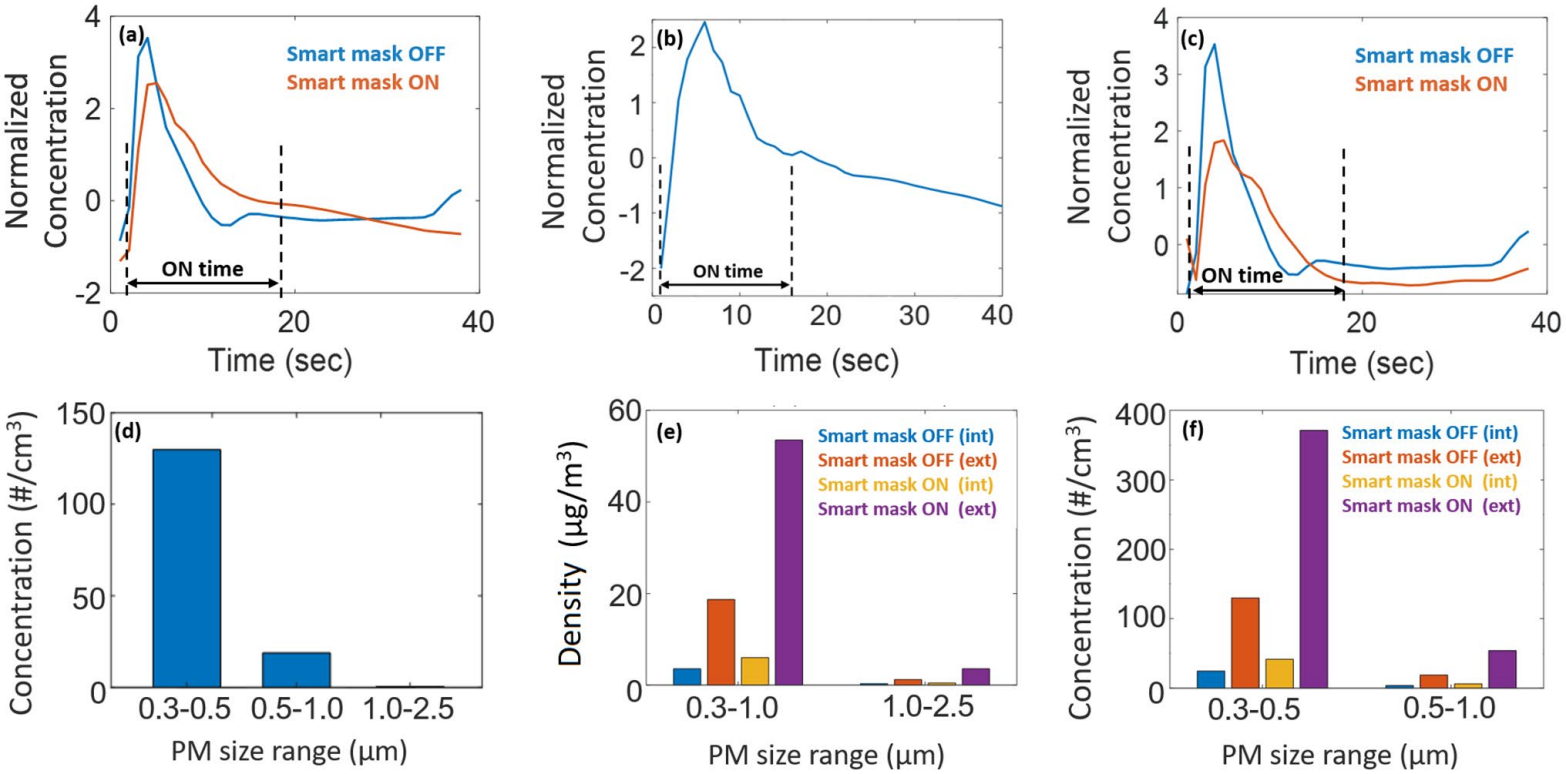
Normalized PM concentration for the particle size range 0.3–$1.0\,\mu \hbox {m}$ near the smart mask (**a**) decreases as the smart mask is switched ON, (**b**) is increased by the aerosol droplets generated by the smart mask itself (self-interference), and (**c**) decreases significantly after subtracting self-interference. (**d**) Typical PM concentration generated by the humidifier (representing activities such as talking, coughing, and sneezing). Comparison of (**e**) time-averaged density, and (**f**) time-averaged concentration for the two PM sensors (int: on the mask, ext: on the ground) as the mitigation module is turned ON and OFF. Plots adapted from^20^.

The observed trends in total PM concentration (1/$\hbox {cm}^3$) and density ($\mu \hbox {g}/\hbox {m}^3$) during these tests were described in our previous work^20^ and are reproduced in Fig. 3 for completeness. Figs. 3a–c show that the time-averaged PM concentration around the mask in the $d=0.3$–$1\,\mu \hbox {m}$ range decreases significantly ($\sim 40\%$) when the mitigation module is enabled. Fig. 3d confirms that the aerosol cloud generated by the humidifier has a relatively uniform size distribution that peaks in the $d=0.3$–$0.5\,\mu \hbox {m}$ range, similar to that generated during daily activities^5,6^. Finally, Fig. 3a–c show a significant increase in PM concentration and density for the “ext” sensor (on the ground) when the mitigation module is turned ON, which confirms the expected aerosol loading effect of the mist spray.

**Figure 4:**
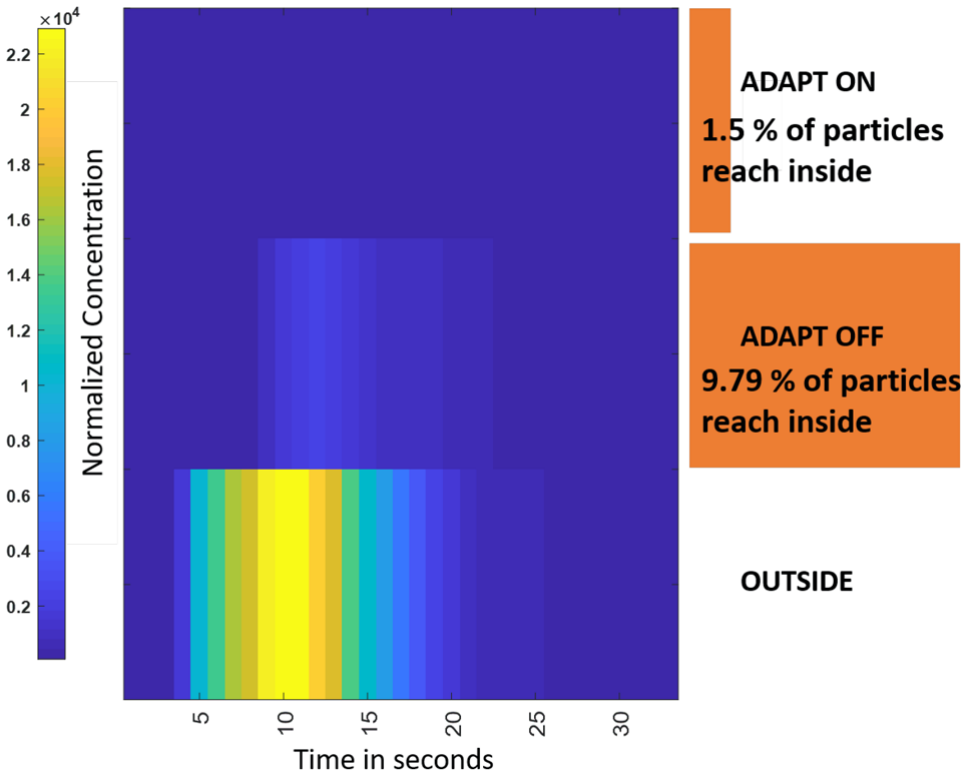
Normalized PM concentration versus time for the particle size range 0.3–$1.0\,\mu \hbox {m}$ in three cases: just outside the mask (bottom), within the mask with the mitigation module OFF (middle), and within the mask with the mitigation module ON (top). The mist generated by the mitigation module reduces the internal PM concentration by approximately $6.5\times$.

The piezoelectric transducer ejects droplets at a specific pressure, thus imparting a significant initial velocity to the mist in the direction facing the mask. To understand the efficiency of this flow in pushing away incoming aerosols from the mask, we used two PM sensors: one placed inside the mask, and the other just outside. Incoming aerosols were generated by a humidifier pointed towards the mask and set at its maximum mist generation rate. We then analyzed the number of particles entering the mask in two scenarios: mitigation module switched ON and OFF, respectively. Note that even though the mask is made from a solid material, in general small particles can easily enter it through air gaps between the mask and face, especially near the nose. For instance, Fig. 4 shows that $\approx 9.79\%$ of the particles that reached the mask found their way inside when the mitigation module was OFF. However, this number decreased to $\approx 1.5\%$ when the module was turned ON. Thus, the mist generator increased the effectiveness of the mask by approximately $6.5\times$.

### Temporal dispersion of aerosol particles

**Figure 5:**
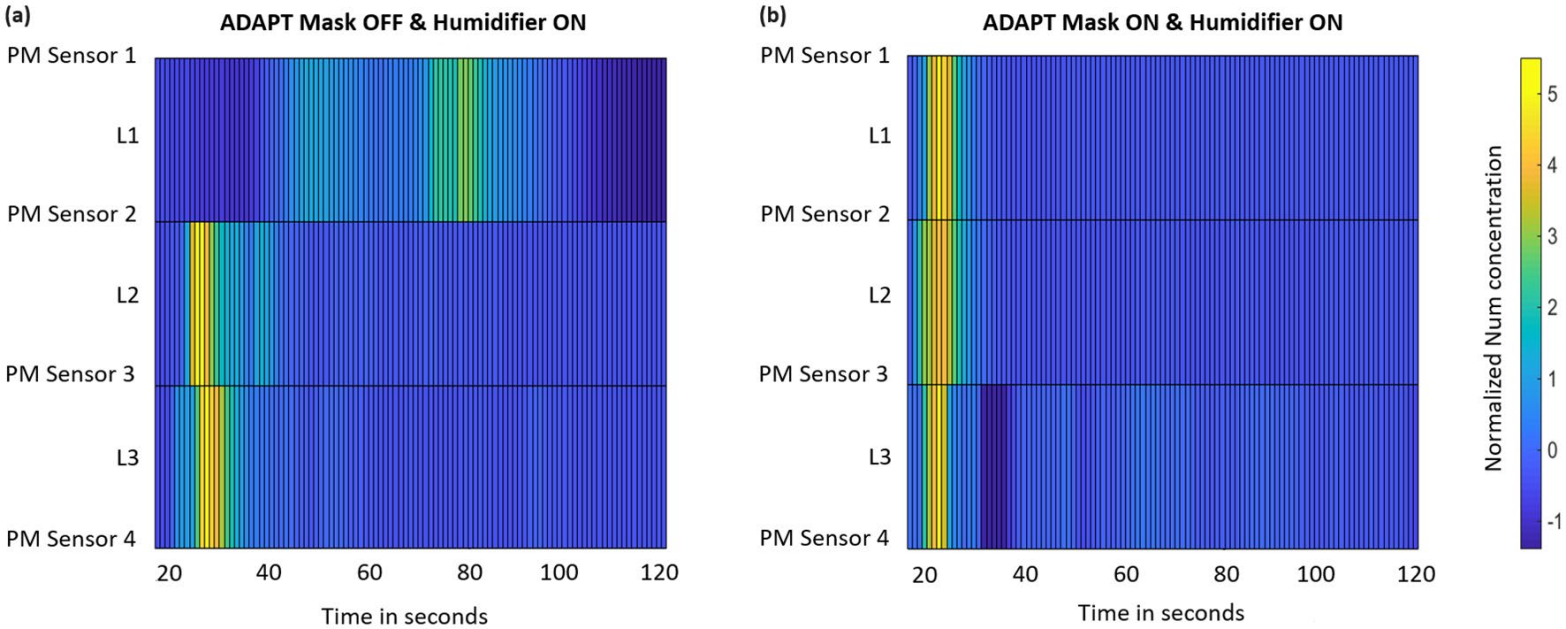
Normalized aerosol concentrations detected by four PM sensors (#1–#4) at different heights as a function of time, interpolated to three different levels ($L_1$, $L_2$, and $L_3$). PM Sensor 1 is placed at 103cm, PM Sensor 2 at 77cm, PM Sensor 3 at 53cm and PM Sensor 4 at 28cm from the ground. (**a**) Only the humidifier is turned ON, showing the presence of an aerosol cloud at level $L_1$ up to $\sim 80\,\hbox {s}$. (**b**) Both the humidifier and mitigation module are turned ON, causing the aerosol cloud to settle to the ground within 20 s.

To visualize the aerosol loading effect with respect to time, we measured PM concentrations over the size range 0.5–$2.5 \,\mu \hbox {m}$ at a sampling rate of 1 sec. Measurements were performed simultaneously at three different levels (i.e., heights) $L_{i}$, where $i\in \{1,2,3\}$, by interpolating the outputs of four vertically-separated PM sensors. The sensors were placed slightly off the line of sight joining the smart mask and the humidifier to minimize their impact on the aerosol cloud. The experiment was started at $t = 0$, and the external humidifier was switched ON at $t = 10\,\hbox {s}$. At $t = 15\,\hbox {s}$, the humidifier was switched OFF and the mask turned ON. Finally, mist generation by the mask was stopped at $t = 20\,\hbox {s}$, and PM readings recorded until $t = 120\,\hbox {s}$. The resulting matrix of PM concentrations is shown in Fig. 5 in two cases: (a) mask OFF (used as a control), and (b) mask ON. These plots allow the dispersion of the aerosol cloud over time to be visualized.

Figure 5a shows the presence of a significant aerosol cloud generated by the humidifier for times up to 80 sec (particularly at level $L_1$). By contrast, Fig. 5b shows no significant aerosol cloud for times $>20$ sec. Additionally, concentrations at the lower levels ($L_2$, and $L_3$) are significantly higher than in the earlier case, again suggesting that aerosol loading by the mist spray greatly reduces the settling time.

### Effects of liquid type on mitigation metrics

**Figure 6:**
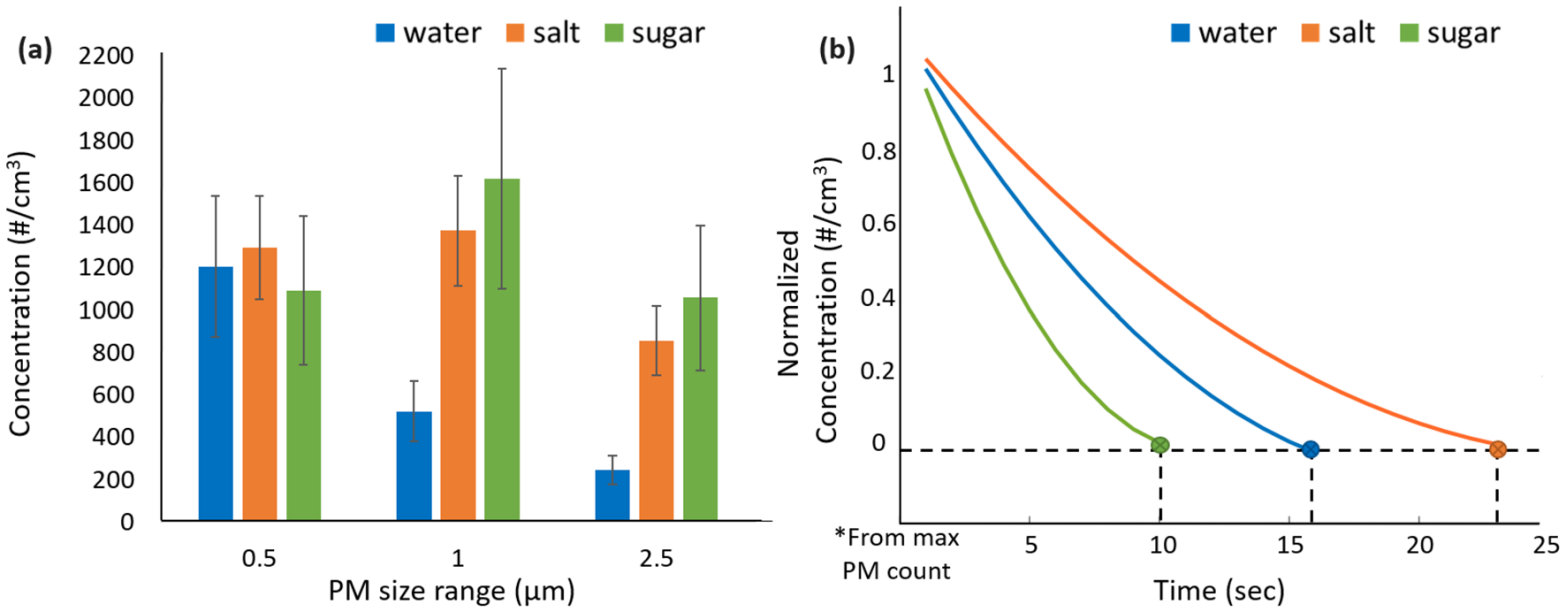
(**a**) Time-averaged PM concentrations over the range 0.5-$2.5\,\mu \hbox {m}$ observed near the mask when tap water, salt solution (brine), and sugar solution (concentration $=125\,\hbox {g/L}$) are used in the mitigation module. (**b**) Comparison of the PM decay rates and settling times for mist generated by the three solutions. The sugar solution results in the shortest settling time.

Aerosol loading experiments were repeated with aqueous solutions of salt (NaCl) and sugar (sucrose) used within the mitigation module instead of tap water. The resulting time-averaged PM concentrations observed near the mask are summarized in Fig. 6a. Both salt and sugar solutions result in significantly larger droplet sizes than pure water, particularly for $d>1\,\mu \hbox {m}$. Such increases in mean droplet size, which are likely driven by the well-known increase in surface tension and viscosity of water solutions for certain solutes (including NaCl and sucrose)^21,22^, may provide users with options for further reducing aerosol settling times.

To further study this effect, we used the same setup as in the previous experiment to measure the time-dependence of number concentration for each solution at PM sensor #4. The normalized values versus time are shown in Fig. 6b. The slopes of these plots are proportional to the settling velocity of the aerosol particles. The data confirms that the observed decrease in small droplet concentration for the sugar solution (see Fig. 6a) results in significantly shorter settling times ($\approx 10\,\hbox {s}$) than for pure water ($\approx 16\,\hbox {s}$). No such decrease is observed for brine: in fact, Fig. 6a shows that the small droplet concentration increases slightly. Since settling time is determined by the smaller droplets, the result is a longer settling time for the brine solution ($\approx 23\,\hbox {s}$) than for pure water.

### Effects of ADAPT on relative humidity (RH)

**Figure 7:**
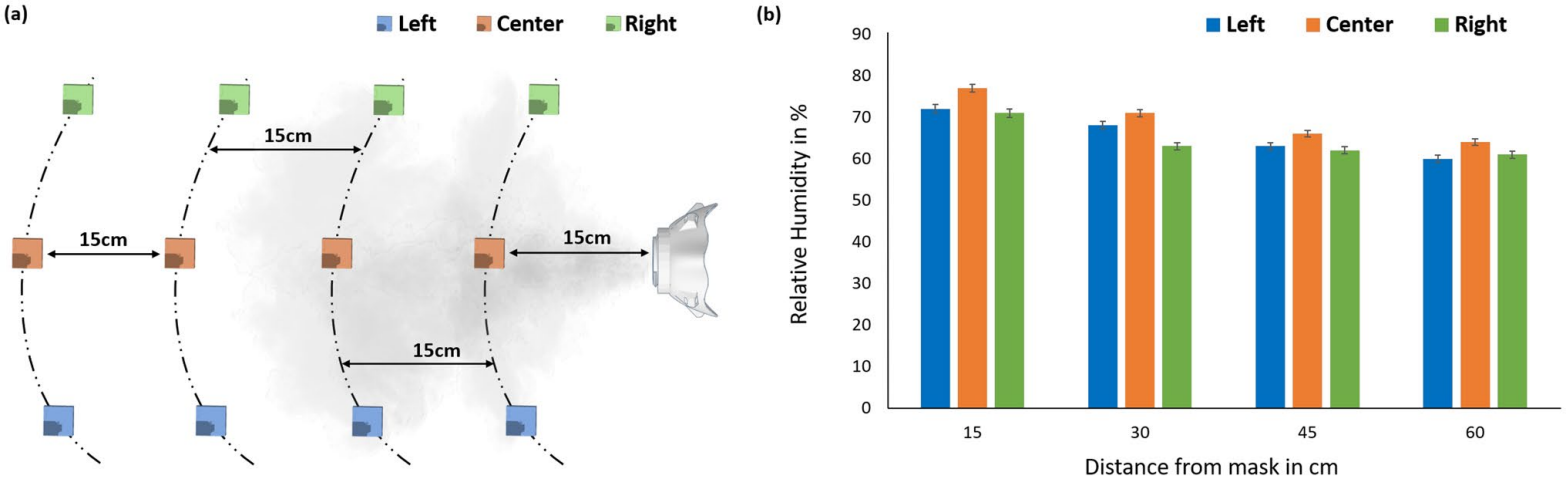
Effects of ADAPT on local RH: (**a**) measurement locations (at distances of 15, 30, 45, and 60 cm from the mask), and (**b**) measurement results. The room temperature was maintained at $23^{\circ }\hbox {C}$.

Research suggests that maintaining higher RH levels can help improve resistance against influenza infections^15,23^. Since ADAPT relies on a water-based mitigation module, we expect its use to provide additional health benefits by increasing local RH levels. We verified this behavior by using a capacitive humidity sensor (DHT11, Adafruit) to measure RH at different locations near the mask (Fig. 7a) when its mitigation module is ON. The results (Fig. 7b) show significant RH increases in all cases compared to the equilibrium RH of the room ($\approx 55\%$). For example, the “central” locations exhibit RH values between 64% (at 60 cm) and 77% (at 15 cm).

## Discussion

We have demonstrated ADAPT, a new active closed-loop “smart mask” paradigm that can both defend the wearer and reduce the spread of airborne pathogens such as SARS-CoV-2. ADAPT is capable of controlling the duration and intensity of mist generation based on its awareness of the current location (e.g., a hospital, quarantine zone, or care facility), ambient conditions (e.g., temperature, humidity, and human occupancy), and health indicators (e.g., age and preexisting conditions). The mask design can potentially be further extended to provide real-time active protection against other airborne hazards including pollutants, dust, and pollen.

The physical design of ADAPT was derived from an open-source mask project, modified to (i) add breathable perforations, and (ii) house a liquid reservoir, piezoelectric transducer, and PM sensor. The perforations are internally shielded using replaceable air filters to further reduce PM concentrations. The experimental prototype was 3D-printed using polylactic acid (PLA). It consists of two main parts, each weighing $\sim$110 gm: (i) the mask itself, and (ii) a belt unit that houses the micro-controller and battery, Tests on volunteers confirmed that the device is comfortable enough for long-term use. Further improvements in the manufacturing process (e.g., using injection molding) and integration of the electronics can reduce system size/weight and eventually eliminate the belt unit.

Test results from a working ADAPT prototype confirm that the mitigation module significantly reduces PM concentrations near the mask via aerosol loading. The use of aqueous salt and sugar solutions increases mean droplet diameter compared to water, and thus has the potential to further reduce PM settling times. Such solutions are also known to have antibacterial properties^24^. In addition, the mask was found to increase local RH, which is beneficial since it enables nasal mucus to filter out pathogens before they enter the respiratory tract^1,23^.

Further work will focus on improving the sensor to provide additional information on pathogen type, thus allowing the mitigation strategy to be appropriately optimized. Laser-induced fluorescence^25^ is promising for this purpose. The on-board algorithm for deploying the mitigation technique can also be improved. For example, artificial intelligence (AI) can be used to determine i) when the module should be turned ON, and ii) the optimum mitigation parameters as a function of location, environmental conditions, health data, and other variables.

## Materials and methods

**Figure 8:**
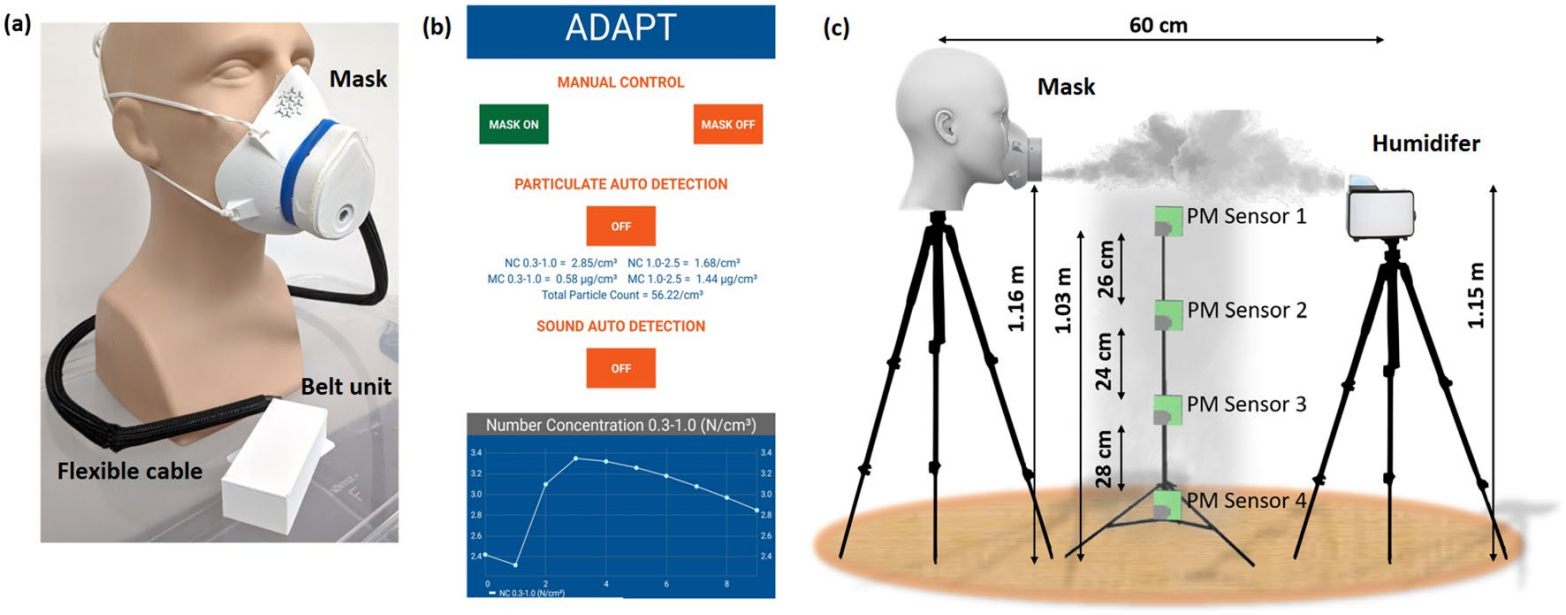
(**a**) Prototype of the ADAPT smart mask. (**b**) Screenshot of the mobile application visualizing data from the ADAPT mask. Manual working mode with ON and OFF buttons can be seen, along with options for automatic and sound-based activation. (**c**) Experimental setup for visualizing the aerosol cloud generated by the humidifier.

### Smart mask

**Figure 9:**
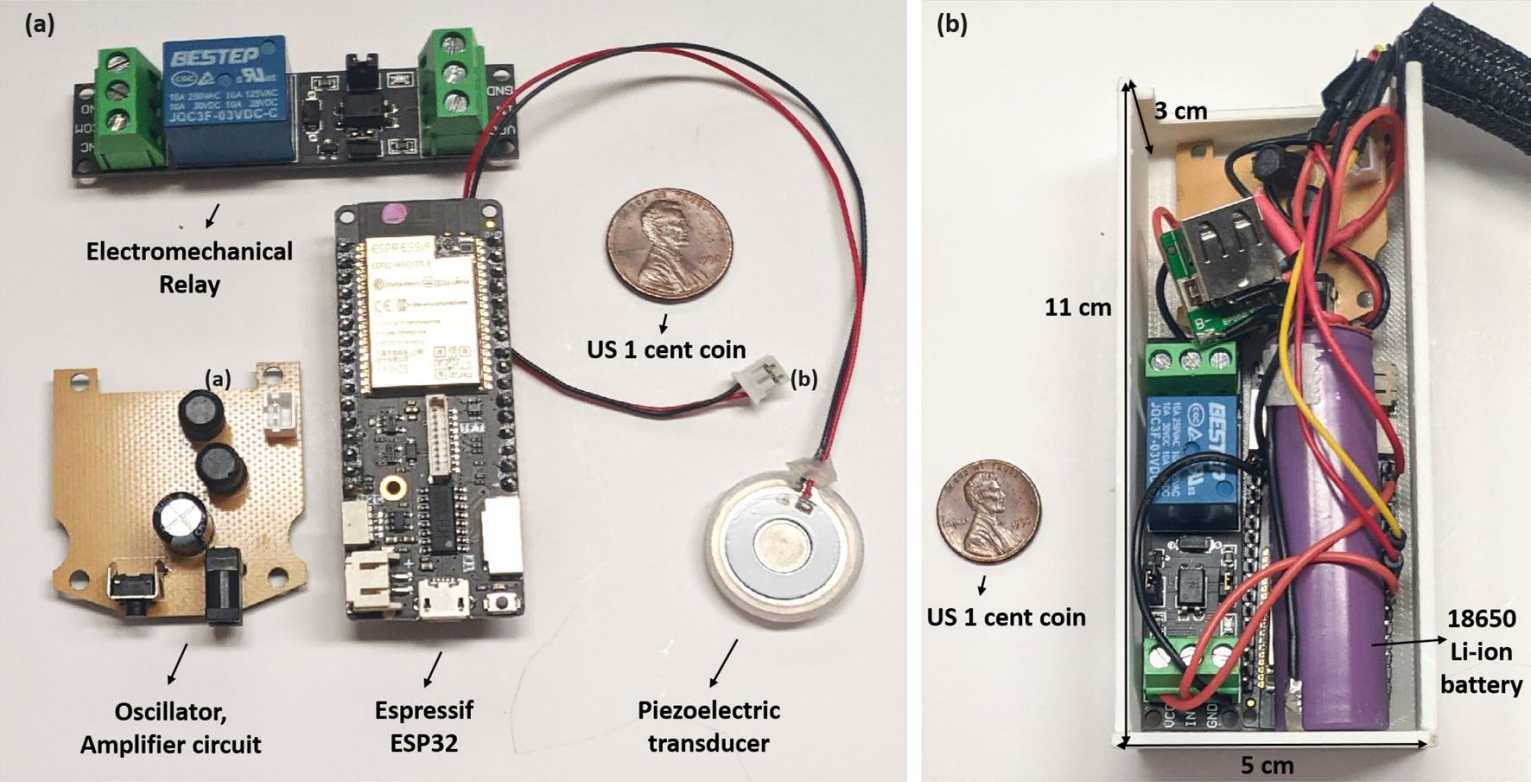
(**a**) The individual components in the belt unit: relay, oscillator, and amplifier circuits for the piezoelectric transducer, the Espressif ESP 32 microcontroller, and the transducer. (**b**) Fully assembled belt unit, including the Li-ion battery with its voltage conversion circuit and a USB port for charging the battery.

The current version of ADAPT consists of two main parts: the *mask unit* and the *belt unit* (Fig. 8a). The former, which is 3D printed, contains the PM sensor (Sensirion SPS30), the liquid reservoir, and the piezoelectric transducer. The liquid reservoir holds up to 65 ml of water. The transducer is secured within the reservoir with a water-tight seal to prevent leaks. It also has an access port on the top for filling/emptying the liquid. The sides of the mask unit include slots for straps that allow stable positioning on the user’s face, while the top has ventilation holes that allow air flow (this is necessary since the body of the mask is entirely solid). The holes are covered by removable multi-layer air filters (placed internally) to minimize aerosol intake. The mitigation module comprises of the transducer, its driver circuits (amplifier, oscillator), and a relay. Apart from the transducer, the rest are housed in the belt unit. A flexible cable, (Fig. 8a) transfers data and power from the belt unit to the PM sensor and transducer. The former also contains the microcontroller and battery, as shown in Fig. 9a,b, respectively.

### PM sensor

The Sensirion SPS30 is an industrial-grade PM sensor (based on laser scattering) that can quantify PM number concentration for the following size “bins”: $0.5\,\mu \hbox {m}$, $1.0\,\mu \hbox {m}$, $2.5\,\mu \hbox {m}$, $4\,\mu \hbox {m}$ and $10\,\mu \hbox {m}$. The sensor uses a small built-in fan for self-cleaning. However, the exhaust from this fan interferes with the motion of the aerosol particles being measured. Adding a small plastic tube to redirect the exhaust reduced this effect. Sensirion provides software that allows the PM sensor to be connected to a computer for data storage. The software also allows for real-time viewing of the collected data, which is initially stored in EDF format and later exported to a spreadsheet for further processing. The sensor can also transfer data to a microcontroller via either $\hbox {I}^2\hbox {C}$ or UART protocol. Considering the length of the cable required to connect the belt unit to the mask, we selected the lower-speed $\hbox {I}^2\hbox {C}$ protocol.

### Mist generator

The water present in the reservoir is converted to mist by a piezoelectric transducer, shown in Fig. 9a. The transducer has two disc-shaped electrodes. The anode, which faces the environment, has a fine mesh in the center; the latter faces the water and also acts as a backplate on which a hollowed-out cathode rests. The hollow area on the cathode allows the mist to pass through the mesh anode into the environment. Fluid pressure in the reservoir is kept low enough to allow surface tension to prevent water leakage through the mesh. The mitigation module generates mist by vibrating the transducer near its resonant frequency (110 kHz) to generate a pressure drop across the mesh. This sudden pressure drop converts the water into mist which then escapes through the mesh. The transducer is driven by an amplifier and an oscillator circuit which are housed in the belt unit. The transducer consumes 300 mA of current when driven at 5 V from a single 2200 mAh Li-ion battery; the corresponding continuous mist generation rate is $\approx 300\,\hbox {ml/h}$. During typical use, the module is turned ON (via a relay) for 30 sec every time high PM counts are detected. Assuming 100 detection events per day, the expected battery life is $\approx 6\,\hbox {days}$.

### Microcontroller

An Espressif ESP32 microcontroller, shown in Fig. 9a, is used to communicate with the on-board PM sensor, control the mitigation module, and provide real-time data to a smartphone. The microcontroller interfaces with the PM sensor via a wired $\hbox {I}^{2}\hbox {C}$ bus. Acquired data is relayed to nearby mobile devices over Bluetooth and accessed via an Android application as shown in (Fig. 8b). The app lets users manually turn the mitigation module ON/OFF, thus overriding the built-in automated control loop. The latter uses a threshold-based algorithm (Fig. 2) to analyze data from the PM sensor and then make decisions on turning the mitigation module ON/OFF. The algorithm also monitors the mobile device’s built-in microphone to detect relevant audio cues and switches ON the mitigation module if necessary (e.g., when a sneeze or cough is detected). The Android app, when connected to the smart mask, also provides real-time PM measurements, as relayed from the PM sensor through the microcontroller.

### Experimental setup

During the experiments, the smart mask was operated in manual mode. Both the on-board PM sensor and the external PM sensors were set to output data once every second. The former was interfaced to the on-board microcontroller, while the latter were interfaced to a laptop computer (via a USB hub) to capture data. The computer was situated far from the experimental setup to ensure that its ventilation fans did not affect airflow around the aerosol dispersion area. All the experiments were manually timed using a stopwatch. An off-the-shelf humidifier was manually triggered to generate aerosol clouds in the $0.3-2.5$ $\mu$m range on-demand, thus replicating the effect of daily activities like talking, coughing, and sneezing. The experimental setup used to determine effects of the ADAPT mask on aerosol concentration, i.e., the results shown in Fig. 3, was as follows. One PM sensor was placed on the mask at the height of 1.6 m, while a second was placed on the ground midway between mask and humidifier. Both sensors were connected to a laptop to acquire data, while the mobile app was used to manually control the mitigation module. The humidifier was turned ON for 15 sec, and the mitigation module on the smart mask was activated for 15 sec immediately after the PM sensor detects a significant local change in PM concentration. The transducer driver circuit was supplied with 12 V for this purpose. The outputs of both PM sensors were then measured for $\approx 160\,\hbox {s}$. The number concentration curves were then plotted until most PM had settled out ($\approx 40\,\hbox {s}$).

The experimental setup used for determining the temporal dispersion of aerosol particles is shown in Fig. 8c. The smart mask and the humidifier were placed on tripods such that they were separated by 60 cm and located 1.16 m and 1.15 m, respectively, above the ground. A vertical stack of four PM sensors was positioned $\approx 25\,\hbox {cm}$ away from the line of sight between the humidifier and smart mask; the resulting sensor-humidifier and sensor-mask distances were both equal to 40 cm. The sensors were glued to a tripod using hot glue and labeled sequentially from top to bottom, with the topmost being PM sensor #1; the optimized sensor-ground heights were 1.03 m, 77 cm, 53 cm, and 25 cm, respectively. A relatively long measurement interval ($\approx 120\,\hbox {s}$) was used to ensure complete settling of the PM. First, both the humidifier and the smart mask were switched OFF for 10 sec. Then the humidifier was switched ON for 5 sec. Finally, the smart mask was switched on for 5 sec. A noticeable change in the aerosol PM count was typically observed starting at $t=16\,\hbox {sec}$, so the measurements shown in Fig. 5 start from this point. The driving circuit for the transducer was connected to a 5 V Li-ion battery during this experiment.

We studied the effects of liquid type on the mitigation technique by using sugar and salt solutions in the reservoir instead of water. The approximate concentrations of both sugar and salt solutions were 125 g/L. To prevent contamination, the reservoir was cleaned with fresh water before each experiment. The driving circuit for the transducer was connected to a 5 V Li-ion battery during this experiment.

Moreover, we studied the effects of the smart mask on relative humidity by using a humidity sensor (DHT11, Adafruit Industries) connected to another ESP32 microcontroller. The sensor was placed at four different distances, namely 15 cm, 30 cm, 45 cm, and 60 cm, and used to measure humidity at three different points in each case (see Fig. 7a). The driving circuit for the transducer was connected to a 5 V Li-ion battery, while the humidity sensor and microcontroller were connected to a separate Li-ion battery pack. All experiments were conducted in a temperature-controlled room. By placing the test setup in a closed plastic box ($2\times 2\times 2\,\hbox {m}$ in size) isolated from nearby air-conditioning vents, we eliminated the effects of air currents. The setup was accessed via a small port ($2\times 0.6\,\hbox {m}$ in size) created in one of the side walls.
